# Public trust and genomic medicine in Canada and the UK

**DOI:** 10.12688/wellcomeopenres.16831.2

**Published:** 2021-07-01

**Authors:** Sarah Savić-Kallesøe, Anna Middleton, Richard Milne

**Affiliations:** 1 Department of Public Health and Primary Care, University of Cambridge, Cambridge, CB2 0SR, UK; 2 Society and Ethics Research, Wellcome Connecting Science, Cambridge, CB10 1SA, UK; 3 Faculty of Education, University of Cambridge, Cambridge, CB2 8PQ, UK

**Keywords:** genomics, public trust, data sharing, Canada, United Kingdom, personalised medicine, personalized medicine, genomic medicine

## Abstract

**Background: **Genomic medicine could improve precise risk stratification, early prevention, and personalised treatment across a broad spectrum of disease. As this reality approaches, questions on the importance of public trust arise. The success of genomic medicine initiatives is influenced by the public’s trust and willingness to engage. Specific social actors influential in the public's trust have been identified by the “Your DNA, Your Say” study, including doctors, researchers, and governments. This paper aims to identify and examine which specific social actors, if any, in Canada and the United Kingdom (UK) are the most trustworthy and influential to engage the public in genomic medicine research.

**Methods: **Using data from the ‘Your DNA, Your Say’ study, logistic regression models and Pearson’s chi-square tests were conducted to explore trust in social actors across Canada and the UK.

**Results: **The results demonstrate Canada and the UK significantly differ in public trust and willingness to donate. Non-profit researchers, domestic doctors, and personal doctors were identified to be the most influential and trustworthy social actors in Canada and the UK.

**Conclusions: **The comparative results indicate that both countries would benefit from engaging the public through doctors and non-profit researchers. The UK could additionally support public trust by engaging with the public through the National Health Service. However, the results suggest that whilst public trust is significant, it may be neither necessary nor sufficient in influencing willingness to donate. Future research could do well to investigate how the importance of public trust compares in countries with lower public trust.

## Introduction

### Genomic medicine and public health

The sharing of patient data, especially genomic data, across a healthcare system is pertinent for the improvement of population screening and patient care. Broadly defined, genomic data refers to an individual’s sequenced DNA ^1^. Genomic medicine, a subset of precision medicine, pertains to using an individual’s genomic data to guide clinical care, including screening, diagnostics, and treatments ^2^. Medical information includes individuals’ demographic profile and medical history of diagnostics, diseases, and treatments ^3^.

At the population level, genomic medicine may revolutionize how we identify and care for populations at high-risk for an array of diseases. A key public health implication of genomic medicine is population risk stratification based on genetic predisposition, allowing for earlier, more precise, and more cost-effective intervention ^4^ and the potential to improve population screening for a spectrum of diseases. Genomic medicine integrated into routine healthcare would afford patients robust disease risk prediction, improved precision and positive predictability value of diagnostics, and medication regimes tailored to their genetic profile ^5^.

Integrating genomic medicine into routine healthcare involves collecting, storing, and analyzing data from the genomes of a large number of individuals. Consequently, many countries have pledged to utilize genomic medicine into healthcare through large-scale sequencing initiatives involving at least 100,000 citizens, including the UK, Saudi Arabia, Turkey, France, and the United States (which aims to a sequence a million genomes) ^6^.

### Public trust

The success of genomic medicine depends on the willingness of the public, as patients and non-patients, to make genomic and health information about themselves available to others. A key factor in this is people’s trust in the government’s and healthcare system’s ability to securely store, disseminate, and analyze data. While difficult to measure, evidence suggests that public trust in government in Anglo countries, including the US, Canada, and the UK, has been generally declining ^7^.

In the UK, the ‘care.data’ initiative announced in 2013 was meant to facilitate health service and provision planning by extracting and pooling patient data from general practitioner offices into a national database ^8, 9^. The default ‘opt-out’ of the programme, which required action to withdraw consent, was met with controversy and public disapproval ^9, 10^. The care.data initiative was abandoned in 2014, which was largely attributed to its failure to gain the confidence of patients, healthcare professionals, and citizens in its procurement of consent and management of data.

Canada faces systemic barriers in patient data sharing, largely due to its decentralized structure; instead of a single national healthcare system, there are 10 provincial, three territorial, and one federal healthcare systems, each providing care for different groups of people, typically based on region ^11^. Each system is the sole owner of its health-related data, including surveillance and patient data ^12^. Currently, there is no pan-Canadian electronic medical data system, whereby a patient’s data is accessible anywhere in the nation ^13^. This results in patients carrying the burden of recording their medical history and presenting it to each new health professional they encounter. However, the lack of successful cooperation between entities results in a patchwork of electronic medical record systems and contributes to patient burden, as well as fading trust in Canada’s healthcare ^7, 14, 15^.

Failed implementations such as this demonstrate the importance of public trust in the success of public health initiatives. The influence of the public’s trust was also evident in the 2009 H1N1 pandemic, when hampered vaccine uptake was attributed to distrust in government and the scientific community, vocalised by groups such as anti-vaccine followings ^16^. Similarly, the efficacy of large-scale lockdowns instated during COVID-19 to prevent the transmission of SARS-CoV-2 was attenuated as public trust and compliance wavered ^17, 18^.

Public confidence in institutional healthcare is also crucial for effective routine patient-doctor relationships. There is evidence indicating that trust in institutional healthcare, such as hospitals and insurance providers, affects individual’s willingness to access care ^17^. There is evidence of a general lack of trust between the public and healthcare system, which may make it more difficult to integrate personal information into routine clinical care ^19, 20^. In addition, marginalised populations facing systemic bias and who may have personal experience with discrimination may develop distrust in institutions and, consequently, access health services disproportionately less ^17^. This is reflected in inequitable outcomes, such as poor adherence to recommendations and worse self-reported health, among marginalised communities ^21– 24^.

Fostering public trust in genomic medicine research poses additional challenges compared to those of other public health initiatives. These differences include that the implications of genomic data extend beyond the patient and to their biological relatives ^25^. Additionally, due to its identifiable nature, DNA information may require exceptional protection ^25^. For these reasons, the integration of genomic medicine into mainstream healthcare must be purposefully and thoughtfully informed by the public voice. At the core of its successful integration is a trusting relationship between the public and the healthcare system.

### Canada and the UK

The “Your DNA, Your Say” (YDYS) study aims to understand the public’s inclination to participate in data sharing based on their willingness to donate their DNA information; it has identified willingness to donate DNA to be related to general public trust in social actors, such as the government, non-profit researchers, and doctors across 22 countries ^26, 27^. These specific social actors were chosen to capture the individuals and institutions involved in collecting and using genomic and health data. Prior publications have investigated how trust and willingness to donate trend across the 22 countries and have examined the Anglo countries as a cluster. These analyses suggest that the public’s trust plays a key role in the population’s willingness to donate DNA; across all countries examined in the YDYS study, overall trust was found to be positively associated with willingness to donate ^28^. This trend is also evident within the grouped analysis of Canada, the USA, Australia, and the UK ^26^.

Previous analyses of YDYS data indicated that Canada and the UK have similarly influential social actors in relation to health data ^26^. This could be linked to their structurally similar universal healthcare systems, similar legal systems, and deep shared historical ties. Given this, we would expect that Canada and the UK would have similar results in public trust and willingness to donate DNA information. Indeed, further analyses have suggested that Canada and the UK may be particularly closely related in terms of how respondents perceive the trustworthiness of data institutions ^28, 29^. A side-by-side comparison of Canada and the UK offer the opportunity to examine whether and how public trust plays different roles in genomic medicine research within two similar countries. In this paper, we investigate which social actors, if any, in Canada and the UK are the most trusted and may be most influential to effectively engage the public in genomic medicine research. We develop a comparative analysis of the UK and Canada to understand the nuances of the public’s trust in social actors involved in genomic medicine research and patient data sharing, such as the government, doctors, and the healthcare system. The implications of such findings would contribute to the evidence base for future informed patient data sharing initiatives. For a larger comparison across countries beyond Canada and the UK, please refer to existing YDYS publications ^28^.

### Objectives

The YDYS study is the source of data for this paper, which includes survey responses from 36,268 individuals across 22 countries, collected from 2017 and onwards. The purpose of the YDYS study is to assess: the public’s attitudes and trust towards donating and sharing of genomic information; the characteristics of those most and least willing to donate; the characteristics of those most and least trusting of government and medical social actors. The research question posed in this paper is “Are there differences in public trust between Canada and the UK and might they affect the public’s willingness to donate DNA information for genomic medicine research?” Considering that the data are cross-sectional and ecological in nature, this investigation is meant to be hypothesis-generating and exploratory rather than conclusive.

Specifically, this paper aims to:

1. Compare proportions of the public’s trust in and the influences of social actors in willingness to donate between Canada and the UK.
2. Assess whether these differences in public trust warrant separate tailored strategies in engaging the public genomic medicine research.
3. Identify potential structures that could explain the variation in public trust and willingness to donate between Canada and the UK.

## Methods

Data from the YDYS study were used for this paper, the methodological details of which have been published separately ^30^. Responses from 36,268 participants were collected through an online survey available in 14 languages across 22 countries. Of those, 2,961 participants were in Canada and 3,406 in the UK, totalling 6,373 participants. Responses were collected by a market research company, Dynata, in 2017. The samples are evaluated to be representative, based on comparisons with national census data of population age and gender structure. Participants were offered a small financial incentive (< £1). The recruitment method did not allow for a known non-response rate. Consisting of 29 questions, the survey ^31^, which can be accessed at YourDNAYourSay.org, takes approximately 15–20 minutes to complete.

### Measure definitions

***Trust.*** Participants’ overall trust and trust in specific social actors were both assessed. Participants were asked whether they generally trust the following social actors: their doctor, any domestic doctor, any international doctor, their country’s government, for-profit researchers, and non-profit researchers. Non-profit researchers included university and publicly funded researchers. For-profit researchers included researchers funded by private companies spanning across a wide scope of industries, such as pharmaceutical, drug discovery, diagnostic, and healthcare technology development. Response options included “Yes”, “No”, and “Unsure”. The “No” and “Unsure” responses were combined for analysis, reflecting the active choice nature of trust discussed above, our interest in investigating the influence of active trust on willingness to donate and to improve the statistical power of tests drawing on respondents who did not actively trust. Participants were defined as “generally trusting” if they reported “Yes" to trusting at least two social actors. In our analysis, we treat trust in certain social actors as proxies for trust in the national healthcare system (trust in any domestic doctor) and trust in the medical profession (any international doctor).

***Willingness to donate DNA.*** Participants were categorised as generally willing to donate if they self-reported willing to donate to at least two of the following: medical doctors, for-profit researchers, or non-profit researchers. Those who did not were categorised as not generally willing to donate.

***Familiarity with genetics.*** Participants self-reported whether they were familiar with DNA, genetics, or genomics. Those who reported familiarity were asked for further detail, and those with knowledge of DNA through family or personal medical history or their work (i.e., genetics counsellor, genetic researcher, etc.) were identified as having ‘personal familiarity’. Those who did not identify being familiar with genetics were categorised as ‘unfamiliar’.

***DNA status.*** Participants were asked whether they regard DNA information to be different from other medical information. Participants were categorised as either regarding DNA information different from other medical information, the same, or unsure. For analysis, ‘same’ and ‘unsure’ responses were combined as we are interested in investigating those who see DNA information as different.

### Analysis

The statistical software Stata, version 16.1, was used to clean the data and conduct analysis. A logistic regression model was created to explore whether public trust is influential in willingness to donate DNA. Pearson's chi-squared tests were performed to identify whether there were statistical differences in the proportions of those willing to donate DNA information or of those who are overall trusting between Canada and the UK. Two logistic regression models, one for each country, were created to investigate how social actors and other factors might influence willingness to donate in Canada and the UK. Factors included familiarity of genetics, perception of DNA information as different from other medical information, and trust in specific social actors. Social actors included the respondent’s doctor, any domestic doctor, any international doctor, their country’s government, a for-profit researcher, and non-profit researcher. Social actors with discordant significance between the country models were further investigated by chi-square tests, exploring whether Canada and the UK significantly differ in proportions who trust these social actors, including doctors and for-profit researchers.

### Ethics

The online survey is fully anonymous. Participants are informed that their consent is given when they choose to click off the landing page and start answering the questions. On the landing page, the purpose of the project is explained as well as what participation involves; participants have a choice at any stage within the survey to stop answering the questions and withdraw. The online project is physically based at the Wellcome Genome Campus with all data collected and stored in encrypted files at the Wellcome Sanger Institute in Cambridge. As part of the conditions of research delivery at this research institution, the project passed ethical review by the Human Materials and Data Management Committee of the Wellcome Sanger Institute (Registration Number: 16/029), as well as legal review to ensure that it was compliant with ethical and legal standards for participant involvement, data collection, and storage.

## Results

### Trust and willingness to donate DNA

As mentioned in the introduction, existing literature has identified a positive association between overall trust and willingness to donate genomic data within Canada and the UK. Pooling the Canadian and UK samples, the logistic regression model in Table 1 suggests that overall trust is significantly associated with willingness to donate; the odds that an individual willing to donate to generally be trusting of social actors is 4.61 times more likely than not generally trusting (95% CI, 4.13-5.15) ( Table 1). In both countries almost half of Canadian and UK respondents report general trust in multiple potential data users (Canada: 48.56%; UK: 53.82%) ^31^.

**Table 1.** Logistic regression: Trust and willingness to donate DNA (pooled).

Canada and the UK (n = 6,367)			
Willing to donate:	*Odds* *ratio*	*P*	*95% * *Confidence* * interval*
Are generally trusting * *(Ref = not trusting or unsure)*	4.61	0.000	(4.13 – 5.15)
Familiar with genetics *(Ref = no familiarity with genetics)*	1.97	0.000	(1.76 – 2.21)
Regard DNA as different from other medical information *(Ref = regarding medical and DNA information to be the same)*	1.53	0.000	(1.37 – 1.71)
Country *(Ref = Canada)*	0.75	0.000	(0.67 – 0.83)
Constant	0.24	0.000	(0.21 – 0.27)

* Defined as participants who trust at least two of the following: their doctor, any domestic doctor, their country’s government, domestic for-profit researchers, or domestic non-profit researchers.

### Comparing Canada and the UK

Chi-square tests were conducted to compare how Canada and the UK differ in willingness to donate DNA and trust ( Table 2). The results indicate there to be a significant difference in overall trust between Canada (48.56%) and the UK (53.82%) ( Table 2). A significantly greater proportion of the Canadian sample (45.96%) was willing to donate compared to the UK (40.49%). Considering there are significant differences in the degree of trust, logistic regression models were developed to investigate the influences of trust on willingness to donate in the Canadian and UK samples separately ( Table 3).

**Table 2.** Differences in public trust and willingness to donate in Canada and the UK.

	Canada (n = 2,961)		United Kingdom (n = 3,406)		
Participants who:	*N*	*%*	*N*	*%*	*P*
Are generally trusting *	1,438	48.56	1,833	53.82	0.000
Are willing to donate DNA	1,361	45.96	1,379	40.49	0.000
Trust their doctor	2,276	76.87	2,638	77.45	0.579
Trust any domestic doctor	1,054	35.60	1,597	46.89	0.000
Trust any international doctor	422	14.25	548	16.09	0.042
Trust non-profit researchers	1,110	37.49	1,124	33.00	0.000
Trust for-profit researchers	345	11.65	330	9.69	0.011
Trust their country’s government	570	19.25	558	16.38	0.003

* Defined as participants who trust at least two of the following: their doctor, any domestic doctor, their country’s government, domestic for-profit researchers, or domestic non-profit researchers.

**Table 3.** Logistic regression models: Trust and willingness to donate DNA.

	Canada (n = 2,961)			United Kingdom (n = 3,406)		
Willing to donate:	*Odds* * ratio*	*P*	*95% Confidence* * interval*	*Odds * *ratio*	*P*	*95% Confidence* * interval*
Trust their doctor	1.78	0.000	(1.43 – 2.21)	1.97	0.000	(1.56 – 2.48)
Trust any domestic doctor	1.53	0.000	(1.24 – 1.87)	1.38	0.001	(1.15 – 1.66)
Trust any international doctor	1.16	0.297	(0.87 – 1.55)	1.13	0.296	(0.90 – 1.44)
Trust non–profit researchers	3.38	0.000	(2.79 – 4.08)	3.91	0.000	(3.26 – 4.69)
Trust for–profit researchers	1.17	0.000	(1.27 – 2.34)	1.31	0.079	(0.97 – 1.78)
Trust their country’s government *(Ref = not trusting or unsure)*	1.47	0.001	(1.17 – 1.86)	1.58	0.000	(1.25 – 1.98)
Familiar with genetics *(Ref = no familiarity with genetics)*	1.72	0.000	(1.46 – 2.04)	2.04	0.000	(1.73 – 2.40)
Regard DNA as different from other medical information *(Ref = regarding medical and DNA* * information to be the same)*	1.42	0.000	(1.20 – 1.68)	1.53	0.000	(1.31 – 1.79)
Constant	0.17	0.000	(0.14 – 0.21)	0.12	0.000	(0.09 – 0.14)

Separate logistic regression models were created for the Canadian and UK samples ( Table 3). Terms predicting willingness to donate included trust in: one’s doctor, any domestic doctor, any international doctor, the country’s government, for-profit researchers, non-profit researchers; familiarity with genetics; and whether they saw DNA information as different from other medical information. All terms were significantly associated with willingness to donate in both country models, except for trust in any international doctor, which was not significant in either country’s model (Canada: OR 1.16, 95% CI 0.87-1.55; UK: OR 1.13, 95% CI 0.90-1.43). Trust in domestic doctors was found to be influential in willingness to donate DNA in both countries ( Table 3); the odds of a willing Canadian to be trusting of any domestic doctor are 1.53 times than to not be willing to donate (95% CI 1.24-1.87) and 1.97 times for a willing individual in the UK (95% CI 1.56-2.48). Trust in for-profit researchers was found to be significant in predicting willingness to donate in the Canadian model (OR 1.73, 95% CI 1.27-2.34) but not the UK model (OR 1.31, 95% CI 0.97-1.78). Doctors and for-profit researchers were of particular interest, suggesting they may contribute to differences in trust and willingness to donate between Canada and the UK. 

***Doctors.*** The proportions of those who trust their doctor, any doctor, and any international doctor were compared between Canada and the UK ( Table 2). Personal doctors hold the highest level of public trust and there is no evidence to suggest that the proportions differ significantly between Canada (76.87%) and the UK (77.45%). The regression models in Table 3 suggest that trust in any domestic doctor is a significant explanatory variable in both Canada and the UK. However, the chi-square results suggest that a significantly greater proportion of the UK sample (46.89%) trust any domestic doctor compared to the Canadian sample (35.60%). Trust in international doctors was not a significant explanatory variable in either model and there was no evidence to suggest there to be a difference in the proportions who trust them between Canada and the UK (Canada: 14.25%; UK: 16.09%).

A chi-square test was run to assess differences in the proportion who trust in their doctor ( Table 4). The oldest group, over 60 years, had significantly higher proportions of trust. Significant differences were also found in trusting domestic doctors across age in the Canadian sample (P=0.003) ( Table 4); the age category with the greatest trust in domestic doctors is the 31–40-year group (40.10%). No significant differences were found in trusting domestic doctors across age categories in the UK sample (P=0.089).

**Table 4.** Differences in trust in doctors across age groups in Canada and the UK.

	Canada (n = 2,961)			United Kingdom (n = 3,406)		
Those who trust their doctor:	*N*	*n*	*%*	*N*	*n*	*%*
30 years and under	451	631	71.47	702	912	76.97
31 – 40 years	452	606	74.59	504	690	73.04
41 – 50 years	404	528	76.52	476	619	76.90
51 – 60 years	447	567	78.84	462	588	78.57
Over 60 years	522	629	82.99	494	597	82.75
	*P = 0.000*			*P = 0.001*		
**Those who trust any domestic doctor:**	*N*	*n*	*%*	*N*	*n*	*%*
30 years and under	236	631	37.40	460	912	50.44
31 – 40 years	243	606	40.10	324	690	46.96
41 – 50 years	198	528	37.50	289	619	46.69
51 – 60 years	181	567	31.92	258	588	43.88
Over 60 years	196	629	31.16	266	597	44.56
	*P = 0.003*			*P = 0.089*		

***For-profit researchers.*** Trust in for-profit researchers was significant in willingness to donate in Canada but not the UK ( Table 3). This discordance of significance was further assessed with chi-square tests and comparisons to trust in non-profit researchers. In both Canada and the UK, the proportion who trust non-profit researchers (Canada: 37.49%; UK: 33.0%) is significantly greater than the proportion who trust for-profit researchers (Canada: 11.65%; UK: 9.69%) ( Table 2). However, these proportions who trust either for-profit or non-profit researchers differ significantly between Canada and the UK.

## Discussion

Identifying social actors who hold the greatest public trust and influence has the potential to support the success of genomic medicine and data sharing initiatives, consequently improving the precision of population risk stratification, earlier preventative care, and, consequently, lightening healthcare burdens. The paper has aimed to understand the nuances in the public’s trust of social actors in Canada and the UK, two countries with relatively high levels of public trust that previous analyses have suggested are similar. Doctors and for-profit researchers were identified to be of interest in the logistic regression models.

Existing YDYS publications suggest overall trust in social actors to be influential in one’s willingness to donate. Considering that the proportions who generally trust social actors significantly differ between Canada (48.56%) and the UK (53.82%), the UK sample was expected to have a higher proportion of those willing to donate ( Table 2). Contrary to expectation, the results suggest that the UK sample has a smaller proportion of participants willing to donate (40.49%) compared to the Canadian sample (45.96%) ( Table 2).

The Canadian sample displays a significantly higher degree of willingness to donate and trust in researchers. Personal doctors hold the highest degree of public trust, followed by non-profit researchers. Trust in personal doctors varies across age, with a greater proportion of older patients (60 years and above) trusting their doctor. Like the UK sample, trust in non-profit researchers appears to have the strongest association with willingness to donate. Based on this evidence, personal doctors and non-profit researchers are the most influential and trustworthy social actors with regards to encouraging the public to donate genomic information for medical research in Canada.

In the UK sample, a significantly greater proportion were generally trusting, trust their own doctor, and trust any domestic doctor, suggesting a strong degree of public trust in the healthcare system. A trend of trust in personal doctors was observed, with the oldest participants (>60 years) displaying the greatest proportion who trust in their doctor. A similar trend in trust for domestic doctors was not found, with no particular age group displaying a significantly different level of trust. Similar to the Canadian sample, trust in non-profit researchers among the UK participants has the greatest association with willingness to donate. However, unlike its Canadian counterpart, trust in for-profit researchers was not found to be significantly associated with willingness to donate.

### Trust in doctors

Trust in three types of doctors were investigated, each representing a different relation with the public. We can understand participants’ trust in any domestic doctors as reflecting their overall trust in the country’s healthcare system, while trust in any international doctor might be understood as a proxy of their trust in the medical profession.

In Canada and the UK, an individual's own doctor holds influential power on participants’ willingness to donate and the highest degree of trust. Trust in international doctors was not found to be influential in willingness to donate in either country. Trust tends to attenuate as social relationships become more distant ^32^, so it is expected that personal doctors would hold greater trust compared to domestic doctors, and for domestic doctors to hold more trust compared to international doctors. The proportions who trust doctors in Canada and the UK are similar, except for trust in domestic doctors; while public trust in domestic doctors is influential in both samples, it is significantly higher in the UK. With regards to the degree of influence on participants’ willingness to donate, personal, domestic, and international doctors held similar levels of influence between Canada and the UK.

***Variation across age.*** Trust in personal doctors significantly varies across age. The age group with the greatest proportion trusting one’s doctor is the oldest group (>60 years), in both Canada and the UK, which is consistent with the positive association between age and trust in doctors previously identified ^33^. Variation in trust across age also exists amongst domestic doctors; the second youngest group (31–40 years) in the Canadian sample had the highest proportion of those who trust domestic doctors, suggesting they may be the most trusting of the healthcare system. However, there was no evidence to suggest a trend across age in the UK sample.

### Trust in domestic researchers

Trust in domestic non-profit researchers was found to be the most influential for willingness to donate in Canada and in the UK ( Table 3). Yet non-profit researchers were the second most trusted social actor in Canada (following personal doctors and followed by domestic doctors) and third most trusted in the UK (following personal doctors and domestic doctors). The difference between Canada and the UK in relative trust between non-profit researchers and domestic doctors further suggests that the healthcare system holds stronger influence on the willingness to donate DNA in the UK.

The influential role of non-profit researchers in public trust in both countries might be associated with their obligation to safeguard and role in analysing patient data ^34^. Similarly, doctors might hold high trust due to their role in applying genomic data findings to medical recommendations ^34^. The stronger influence of trust in non-profit researchers over personal doctors in both countries might highlight the public’s interest in the safeguarding and analysis of their data.

Why is it that trust in domestic non-profit researchers appears more influential in willingness to donate than trust in domestic for-profit researchers? One explanation may be the importance of trust in, and alignment of, motives ^35^. Thus, for non-profit research, which is often founded on principles of altruism or solidarity, it may be more important that there is a perceived alignment of values between the research population and the researcher. The donation of data to non-profit researchers, relies on faith that non-profit researchers will act in the interests of the public good. Conversely, a general lack of public trust and engagement has been observed for for-profit research initiatives that use patient data, yet trust seems less influential in shaping the willingness to donate to these actors ^36, 37^. It also appears that the purpose of data sharing influences the public’s trust of for-profit researchers; sharing health data with commercial companies for patient rather business purposes may be more acceptable to members of the public ^38^. By focusing healthcare and commercial partnerships on patient-centered benefits and data protection, further public trust in for-profit researchers may be garnered.

These findings suggest that, in the development of public genomic medicine initiatives, value alignment between social actors and the public is influential. Moreover, trustworthiness of these initiatives may be reliant on their being guided by non-profit rather than for-profit initiatives.

### Implications

The findings presented here have implications for the development of policy and practice in engaging publics with genomic medicine research. First among these is the role of doctors and the health system as the interface between the public and genomic medicine initiatives. Doctors are a key social actor and potential leverage point in engaging the public in genomic medicine research as they, by professional nature, often act as an interface between the public and the healthcare system. However, there are complications in engaging the public through personal doctors. The most pertinent being that not everyone has a designated doctor (‘family’ doctor), and that those who are more disadvantaged are less likely to have a doctor ^33, 39^. If engagement methods rely on patients receiving information from their doctor, and if discrepancies exist in how primary care is accessed exists, this will systematically disfavour those who are already more disadvantaged. The second consideration is that depending on individual doctors to inform their patients of genomic medicine research in addition to their duties within the limited appointment time may be infeasible and burdensome. Lastly, relying on individual doctors to disseminate information will inevitably foster inconsistency; it would also be impractical to guide efforts pursued by individual doctors rather than to steer a single, composite effort by the healthcare system.

Considering that public trust in the healthcare system is a distinguishing feature between Canada and the UK, policy decisions surrounding the implementations of genomic medicine might reflect the social actors that garner the most trust. The UK might best encourage public trust through individual doctor-patient relations. Considering the high level of public support for, and satisfaction with, the National Health Service (NHS) ^40^, mass-media campaigns carried out by the NHS could contribute to establishing public trust. Comparatively, Canada might more effectively focus on patients’ relationships with personal doctors and with non-profit researchers. This might involve doctors informing patients how their genomic information could affect medical recommendations.

### Necessity vs. sufficiency of public trust

However, it is also important to recognise that our findings show that while public trust is influential, it may not always be necessary. The public regularly interacts with social actors with whom they do not share values with or who are not widely trusted. The nuances of trust also raise questions regarding its sufficiency for willingness to donate. The evidence suggests that the Canadian public is generally more trusting of both non-profit and for-profit researchers. This could be explained if the Canadian sample were more generally trusting. However, the Canadian sample has a smaller proportion who trust social actors overall. Whilst counterintuitive, there are likely other factors influencing trust. One important aspect is that trust is far more nuanced than we can capture in this study. Trust is associated with increased odds to willingness to donate and there are significant differences in the degrees of trust across Canada and the UK. But, as demonstrated in the results, high levels of public trust do not guarantee high levels of willingness to donate; people are willing to donate even when they do not trust, and it cannot be guaranteed that those who do trust will also be willing to donate. Public trust is not sufficient for willingness to donate. Overall trust may not be determinant in willingness to donate, but trust in the social actors involved in the collection, management, storage, and application of one’s genomic information is influential.

### Limitations

A key vulnerability of this study is reductionism. Trust is complex, dynamic, and context dependent, making it difficult to reflect through an exploratory quantitative study. Trusting multiple specific social actors does not necessarily equate to having a high level of general trust. This study focused on identifying social actors who hold the public’s trust. In the exploration of active trust, responses indicating distrust and uncertainty were pooled during statistical analysis. By doing so, this study did not identify social actors who harbour active distrust. Further investigations could compare levels of public trust and distrust of social actors. Identifying characteristics that make social actors more or less trustworthy would be pertinent in the implementation of initiatives that rely on high public trust to be effective. Considering there are no previous publications on the topic of public trust and attitudes towards genomic medicine research using a global survey, evaluating validity by comparing to similar studies is not feasible. Additionally, using an online survey as the method of data collection is limited in that non-response rates are unknown and populations with poor access to the internet are not represented. Lastly, by nature of this study being of cross-sectional design, the questions posed are exploratory in nature; the results speak only to associations and are not conclusive.

## Conclusion

Genomic medicine has the potential to improve precise risk stratification, early prevention, and specialised treatment. However, the public’s trust is consequential for its efficacy. The aim of this research was to investigate how Canada and the UK differ in willingness to donate DNA and how willingness is influenced by trust in social actors. Evidence suggests that public trust in Canada and the UK differ enough to justify tailoring separate methods for engaging the public in genomic medicine research; the UK could benefit more from a mix of personal doctor and healthcare system engagement, whereas Canada could benefit from a method which focuses on engaging the public through their doctors. Both countries could gain from involving non-profit researchers, as trust in them is the most strongly associated with willingness to donate in Canada and the UK. Specific examples could include providing opportunities for researchers to talk about their work and demonstrate why and how they use data. Additionally, this could include offering opportunities for public audiences to engage with researchers in deliberative processes that allow them to question and discuss data sharing topics. Whilst the evidence suggests that trust may not be necessary nor sufficient in the public’s willingness to donate, it also suggests trust is hugely influential. Future research could do well to investigate how the necessity and sufficiency of public trust might vary across countries with low and high overall public trust.

## Data availability

### Underlying data

Open Science Framework: Your DNA Your Say data file. https://doi.org/10.17605/OSF.IO/ZPFGM ^31^.

This project contains the following underlying data:

- YDYS dataset for sharing.csv
- DataDictionary.csv

### Extended data

Open Science Framework: Your DNA Your Say data file. https://doi.org/10.17605/OSF.IO/ZPFGM ^31^.

This project contains the following extended data:

- Word Version GA4GH Survey.docx

Data are available under the terms of the Creative Commons Attribution 4.0 International license (CC-BY 4.0).
