# Supplementary material for: Public trust and genomic medicine in Canada and the UK
Source: Wellcome Open Res. 2021 Jul 1;6:124. Originally published 2021 May 20. [Version 2] doi: 10.12688/wellcomeopenres.16831.2 (PMC8215560; doi:10.12688/wellcomeopenres.16831.2)
Supplement: Peer review history

# Peer review: Reviewer response for version 2

**DOI:** 10.21956/wellcomeopenres.18798.r44682

**Published:** 2021-07-09

**Authors:** Mavis Machirori

**Affiliations:** 1 Ada Lovelace Institute, London, UK

No further comments from me as the authors addressed some of the higher level concerns around what trust might be like or its drivers in different countries.

Is the work clearly and accurately presented and does it cite the current literature?

Yes

If applicable, is the statistical analysis and its interpretation appropriate?

I cannot comment. A qualified statistician is required.

Are all the source data underlying the results available to ensure full reproducibility?

Yes

Is the study design appropriate and is the work technically sound?

Yes

Are the conclusions drawn adequately supported by the results?

Yes

Are sufficient details of methods and analysis provided to allow replication by others?

Partly

Reviewer Expertise:

Diversity of data sets, genomics, data sharing, data governance, engagement

I confirm that I have read this submission and believe that I have an appropriate level of expertise to confirm that it is of an acceptable scientific standard.

# Peer review: Reviewer response for version 2

**DOI:** 10.21956/wellcomeopenres.18798.r44720

**Published:** 2021-07-01

**Authors:** Alessandro Blasimme

**Affiliations:** 1 Health Ethics and Policy Lab, Department of Health Sciences and Technology, ETH Zürich, Zürich, Switzerland

Version 2 is fine for me.

Is the work clearly and accurately presented and does it cite the current literature?

Yes

If applicable, is the statistical analysis and its interpretation appropriate?

I cannot comment. A qualified statistician is required.

Are all the source data underlying the results available to ensure full reproducibility?

Yes

Is the study design appropriate and is the work technically sound?

Yes

Are the conclusions drawn adequately supported by the results?

Yes

Are sufficient details of methods and analysis provided to allow replication by others?

Yes

Reviewer Expertise:

Ethics

I confirm that I have read this submission and believe that I have an appropriate level of expertise to confirm that it is of an acceptable scientific standard.

# Peer review: Reviewer response for version 1

**DOI:** 10.21956/wellcomeopenres.18566.r44006

**Published:** 2021-06-28

**Authors:** Mavis Machirori

**Affiliations:** 1 Ada Lovelace Institute, London, UK

This is an important area of research as noted by the recent and numerous incidents where issues of public trust have been raised. The paper is engaging and provides important points to reflect on for all those embarking on genomic medicine. Importantly, the role of public engagement in genomics is a key conclusion. The following are some minor points to address:

- Typo in Aim 2 (engaging the public in genomic medicine).
- Regarding trust in any domestic/international doctor – would it be possible to critique some of the issues around trying to measure trust with questions such as any domestic doctor/international doctor across the likelihood of people actually accessing said doctors e.g. how likely is it that people in the UK used or accessed other domestic or international doctors to make this question meaningful, rather than hypothetical? Are there any explanations why these differences may exist between the UK and Canada, additionally? 
- If I understand correctly, while more UK participants trusted domestic doctors, in fact, their willingness to donate by this factor was less than the Canadian participants. Does this change the significance attributed to the chi square figures given in Table 2?
- If it is the convention of this journal, then the pre-set level of p-values that were used to calculate significance should also be added for completeness.
- I think the N and n are reversed? N is usually for population and n for sample size, and if I understand, n/N is the sample responding in a certain way out of the entire study sample (e.g. Table 4 ages).
- While not the focus of this paper, could authors comment on the potential limitations of pursuing or implementing genomic medicine especially of personalization, regarding unequal benefits that may arise (e.g. homogenous data sets; donating data does not mean reaping benefits). This is so that any initiatives for public engagement with genomics must also be honest about the potential limitations of genomic medicine as it currently stands.
- One question around the demographics which is alluded to but not explored further is the potential of demographic differences. If this is not reported elsewhere, it will be helpful to understand any differences in the populations responding to the questionnaire to further provide recommendations of tailored trust-increasing interventions. This is especially important given different sections of the community will have different interactions and reasons for trusting. Without this, there will be some normative assumptions about a fairly distributed health care system and the amount of trust directed at the system by different sections of the community.
- Is there anything in the role of government that may lead to lowered trust (e.g. by age also)? While some UK examples are given, there is not much on the Canadian perspective.
- Finally, as part of the recommendations, it would be helpful to explain a little more about what a social license in this area might look like, or what conditions might help it along.

Is the work clearly and accurately presented and does it cite the current literature?

Yes

If applicable, is the statistical analysis and its interpretation appropriate?

I cannot comment. A qualified statistician is required.

Are all the source data underlying the results available to ensure full reproducibility?

Yes

Is the study design appropriate and is the work technically sound?

Yes

Are the conclusions drawn adequately supported by the results?

Yes

Are sufficient details of methods and analysis provided to allow replication by others?

Partly

Reviewer Expertise:

Diversity of data sets, genomics, data sharing, data governance, engagement

I confirm that I have read this submission and believe that I have an appropriate level of expertise to confirm that it is of an acceptable scientific standard, however I have significant reservations, as outlined above.

# Peer review: Reviewer response for version 1

**DOI:** 10.21956/wellcomeopenres.18566.r44003

**Published:** 2021-06-18

**Authors:** Alessandro Blasimme

**Affiliations:** 1 Health Ethics and Policy Lab, Department of Health Sciences and Technology, ETH Zürich, Zürich, Switzerland

The paper reports on the findings of the "Your DNA, your say" (YDYS) study on the public's attitudes towards sharing DNA data (Canada and UK). The topic is relevant to current debates about data sharing, in bioethics and beyond. 

 The paper is based on sound empirical methods, presents interesting interpretations of the findings and offers a valuable contribution to the debate. 

 My only suggestions are:

- To clarify whether DNA data sharing is discussed in the context of research (as it looks like) as opposed to public health and clinical use. 
- To clarify whether participants were asked to express their views in relation to domestic as opposed to international data sharing. 

Is the work clearly and accurately presented and does it cite the current literature?

Yes

If applicable, is the statistical analysis and its interpretation appropriate?

I cannot comment. A qualified statistician is required.

Are all the source data underlying the results available to ensure full reproducibility?

Yes

Is the study design appropriate and is the work technically sound?

Yes

Are the conclusions drawn adequately supported by the results?

Yes

Are sufficient details of methods and analysis provided to allow replication by others?

Yes

Reviewer Expertise:

Ethics

I confirm that I have read this submission and believe that I have an appropriate level of expertise to confirm that it is of an acceptable scientific standard.

# Response

**Published:** 2021-06-25

**Authors:** Sarah Savić-Kallesøe

**Affiliations:** University of Cambridge, Canada

Thank you, Dr. Blasimme, for your time and expertise during this review. Your feedback has been reviewed and integrated into the newest version of the manuscript, including:

 - clarification of the domestic, rather than international, nature of data sharing (discussion); and

 - clarification of the research, rather than clinical, purposes of genomic data sharing within the context of this paper. 

 Thank you, again, for you help.

# Peer review: Reviewer response for version 1

**DOI:** 10.21956/wellcomeopenres.18566.r44004

**Published:** 2021-06-10

**Authors:** Ruth Horn

**Affiliations:** 1 The Ethox Centre and Wellcome Centre for Ethics and Humanities, Nuffield Department of Population Health, University of Oxford, Oxford, UK

This paper makes an important contribution to the understanding of ethical, social and policy issues around genomic medicine by exploring which specific social actors (doctors, researchers and governments) are the most trustworthy and influential to engage the public in genomic medicine. Using data from the “Your DNA, Your Say” study, this paper focuses on the UK and Canada. Non-profit researchers, domestic doctors, and personal doctors were identified to be the most influential and trustworthy social actors in Canada and the UK.

 The authors include links to the database, however, I was unable to open these as the link seemed to be invalid. Maybe the authors could check the link again.

 I have a few minor comments, which, I hope, may help the authors to specify a few more details:

- The reasons the authors provide as to why they focus on the UK and Canada could be further developed. The authors state that they focus on them because of both having comparable universal health care systems, participating in the YDYS study and being Anglo-Saxon countries. These characteristics also apply to other countries and the particularities of why to focus on the UK and Canada could be further developed.
- Furthermore, the specific role of the NHS with regard to public trust in the UK context could be emphasised in the paper (see reports and surveys from the King’s Fund).
- Social actors are defined in the paper as doctors, researchers and governments. Maybe the authors could give a more precise definition of what it means to be a social actor, and why they chose to limit their discussion on doctors, researchers and governments.
- The use of the definition of 'Anglo-Saxon countries' is somehow problematic as US and Canadian populations cannot be considered as predominately 'white Saxons'. ‘Anglo-American countries’ or ‘Anglo countries’ may be more appropriate.
- P. 8 The discussion of public trust towards non-profit versus commercial researchers could be elaborated a little more, and further literature discussing these differences with regard to trust may be added.
- P.9 In the conclusion, the authors state: ‘Both countries could gain from involving non-profit researchers, as trust in them is the most strongly associated with willingness to donate in both countries.’ The authors could add a more explicit statement about what this would mean for public engagement activities, namely the importance of involving non-profit researchers in the public engagement.

Is the work clearly and accurately presented and does it cite the current literature?

Yes

If applicable, is the statistical analysis and its interpretation appropriate?

I cannot comment. A qualified statistician is required.

Are all the source data underlying the results available to ensure full reproducibility?

Yes

Is the study design appropriate and is the work technically sound?

Yes

Are the conclusions drawn adequately supported by the results?

Yes

Are sufficient details of methods and analysis provided to allow replication by others?

Yes

Reviewer Expertise:

ethical issues with regard to the implementation of genomic medicine in different socio-cultural contexts

I confirm that I have read this submission and believe that I have an appropriate level of expertise to confirm that it is of an acceptable scientific standard.

# Response

**Published:** 2021-06-25

**Authors:** Sarah Savić-Kallesøe

**Affiliations:** University of Cambridge, Canada

Thank you Dr. Horn for your time and expertise. Your feedback has been reviewed and integrated into the newest version of the manuscript, including:

 - updating "Anglo-Saxon" countries to "Anglo" countries (introduction);

 - providing further context of the public's support of the UK NHS (using the King's Fund 2019 survey) (discussion);

 - clarification of social actors (introduction); and

 - elaboration of the role of for-profit researchers (discussion).

 Thank you, again, for your help.
